# Primary Immunodeficiency Disorders and Inborn Errors of Immunity in Saudi Arabia: Current Evidence on Epidemiology, Clinical Impact, and Healthcare System Challenges

**DOI:** 10.3390/healthcare14182996

**Published:** 2026-09-14

**Authors:** Meyad Alkarni

**Affiliations:** Department of Clinical Laboratory Sciences, College of Applied Medical Sciences, King Saud University, Riyadh 11433, Saudi Arabia; malkarni@ksu.edu.sa

**Keywords:** primary immunodeficiency disorders, inborn errors of immunity, epidemiology, healthcare systems, consanguinity

## Abstract

The term inborn errors of immunity (IEI) is now widely used to describe many conditions historically known as primary immunodeficiency disorders (PIDs), comprising a heterogeneous group of rare diseases that affect immune system development and/or function and increase the risk of inflammation, infection, immune dysregulation, and malignancy. In Saudi Arabia, the clinical impact of PID/IEI is particularly significant owing to the high consanguinity rates, delayed diagnosis, variable access to specialized immunology services, and limited national records. This narrative review summarizes the available literature on PID/IEI in Saudi Arabia, emphasizing the epidemiology, clinical impact, diagnostic delay, treatment access, healthcare system challenges, and evidence gaps. Relevant literature was identified through searches of PubMed, Scopus, Web of Science, and Google Scholar, supplemented by regional and grey literature, where applicable. The available evidence suggests that PID/IEI remains underrecognized in Saudi Arabia, with published data largely derived from single-center studies, selected cohorts, and hospital-based registries. The current literature highlights important recurring themes, including the significance of consanguinity, the high reported incidence of severe combined immunodeficiency (SCID) in selected studies, timely diagnosis, registry data, and the value of strengthening screening and referral pathways. However, evidence regarding treatment costs, long-term morbidity, quality of life, and regional disparities in access to care remains scarce. Establishing national PID/IEI registries, expanding newborn screening, enhancing primary-care physicians’ awareness, improving genetic diagnostic capacity, and developing clearer referral pathways are important priorities to improve early diagnosis, patient outcomes, and healthcare system preparedness in Saudi Arabia, prioritized by healthcare authorities under Saudi Vision 2030.

## 1. Introduction

### 1.1. Overview of Inborn Errors of Immunity and Primary Immunodeficiency Disorders

The classification and terminology of primary immunodeficiency disorders have changed considerably in recent years. Many conditions previously described as primary immunodeficiency disorders (PIDs) are now grouped under the broader term inborn errors of immunity (IEI), reflecting a wider clinical spectrum of immune dysregulation and malignancy beyond recurrent infections alone [1,2,3]. Since its original definition in 1952, extensive research has been conducted on PIDs, ranging from relatively mild IgA deficiency to severe forms such as severe combined immunodeficiency (SCID) [4]. PIDs involve defects in both adaptive and innate immunity [5]. Although these conditions have historically been described as PIDs, characterized by recurrent infections, IEI may also present with autoimmunity, autoinflammation, lymphoproliferation, allergy, and malignancy. Crucially, over 50% of IEIs are identified in patients beyond the age of 25 years, and approximately 20% do not exhibit infection [6,7,8].

Throughout this review, the term PID/IEI is used to reflect a broader and evolving spectrum, encompassing both childhood-onset genetic disorders and later-onset presentations. Based on the pathogenesis and clinical features, the International Union of Immunological Societies (IUIS) has classified IEIs into major categories based on the affected immune pathway and clinical phenotype (Table 1) [8,9,10]. Although the current IUIS classification provides an important clinical framework, this review focuses on the available evidence regarding the epidemiology, clinical impact, diagnostic delay, treatment, and healthcare system challenges of PID/IEI in Saudi Arabia.

The classification of IEI is regularly updated by the IUIS, consistent with the growing number of genetic defects being identified and the expanding clinical spectrum of these disorders [9,11]. Progress in molecular diagnostics, particularly in next-generation sequencing, has enhanced the detection of monogenic causes and has contributed to a deeper understanding of immune deficiency phenotypes [12]. Several factors make Saudi Arabia an important setting for assessing PID/IEI, including the high prevalence of consanguinity, a corresponding burden of severe autosomal recessive disorders, and a healthcare system in which immunologists and specialized genetic experts are largely concentrated in tertiary care facilities [13,14]. However, much of the available Saudi evidence comes from tertiary care cohorts and selected screening studies; therefore, this review describes the current evidence and its gaps, rather than providing nationally representative estimates.

### 1.2. Genetic Factors

Many PIDs/IEIs are genetic disorders with variable inheritance patterns, including autosomal recessive or X-linked traits [11,15,16]. Globally, many cases remain undiagnosed, underdiagnosed, or unreported. This gap is more pronounced in the Middle East [15]. In Saudi Arabia, the high prevalence of consanguinity plays a major role in the inheritance of IEIs and contributes to the high prevalence of severe inherited immunodeficiencies. Reported genetic defects include mutations in *IL2RG*, *RAG1/2*, and *ADA* [17]. However, despite growing genetic insights, evidence from Saudi Arabia remains fragmented and has predominantly been derived from tertiary care cohorts, thus limiting the generalizability of the current findings and underscoring the need for a focused review of the available evidence. This review aimed to synthesize the available evidence on PID/IEI in Saudi Arabia, highlighting key aspects such as epidemiology, clinical impact, diagnostic delays, access to treatment, challenges within the healthcare system, and areas in which evidence is lacking. Accordingly, this review focuses on the evidence pertinent to Saudi Arabia and outlines actionable priorities for enhancing early diagnosis, developing registries, implementing newborn screening, improving referral pathways, ensuring specialized care, and fostering future research.

## 2. Literature Search and Narrative Synthesis

A literature search was conducted for this narrative review to identify relevant publications on primary PIDs, increasingly known as IEIs, in Saudi Arabia. This search aimed to identify evidence on epidemiology, clinical presentation and impact, diagnostic delay, genetic findings, treatment access, and healthcare system challenges. The PubMed, Scopus, and Web of Science databases were searched from inception up to 12 June 2026. Google Scholar was also searched to identify additional relevant studies. Only English language publications were included. Search terms included “Primary immunodeficiency,” “primary immunodeficiency disorders,” “inborn errors of immunity,” “Saudi Arabia,” “Kingdom of Saudi Arabia,” “consanguinity,” “SCID,” “severe combined immunodeficiency,” “newborn screening,” “diagnostic delay,” “registry,” “genetic testing,” and “healthcare challenges.” Search terms were adapted for each source, combined using the Boolean operators AND and OR. Studies addressing the epidemiology, clinical aspects, diagnosis, genetic findings, treatment, outcomes, and healthcare delivery of PID/IEI in Saudi Arabia were included. Selected studies from Gulf Cooperation Council (GCC) countries and international literature were used only to contextualize the Saudi findings; they were not treated as direct evidence of the Saudi setting. Publications were excluded if they were not relevant to PID/IEI or did not provide clinical, epidemiological, genetic, treatment, or healthcare-related information applicable to the Saudi or regional context. Relevant grey literature was obtained from publicly available Saudi Ministry of Health reports, registry reports, and national healthcare system documents. National administrative and routinely collected healthcare data were also sought; however, no publicly available PID/IEI-specific national datasets were identified throughout this search. The findings were synthesized narratively, with Saudi evidence distinguished from contextual GCC and international evidence and interpreted in light of the study design, setting, population, major limitations, and remaining evidence gaps.

## 3. Epidemiology of PID/IEI in Saudi Arabia and Regional Context

The epidemiology of PID/IEI in Saudi Arabia has not yet been fully characterized, primarily because of the reliance on data from hospital registries, individual center studies, and selection of patient groups rather than a nationwide registry. However, the available literature suggests that severe inherited forms of PID/IEI are common in Saudi cohorts, particularly given the high rates of consanguinity and prevalence of autosomal recessive forms. Consequently, findings from Saudi data should be analyzed cautiously, as the published figures may represent referral trends and experiences from tertiary care rather than reflect the true prevalence in the general population.

Studies in Saudi Arabia have reported a notable burden of severe PID/IEI. Al-Saud et al. reported a prevalence estimate of 7.2 per 10^5^ Saudi children, with combined cellular and humoral immunodeficiencies being the most frequent category in that tertiary-care cohort [18]. The King Faisal Specialist Hospital & Research Centre (KFSHRC) database included 502 patients with PIDs; combined immunodeficiencies represented the largest group, and positive family history and consanguinity were reported in many patients [18]. In one systematic review, Ahmed et al. also reported combined immunodeficiencies as the predominant PID/IEI category among Saudi children in their systematic review [19].

Newborn screening and regional studies further support the importance of the severely inherited forms in Saudi Arabia. For example, Al-Mousa et al. reported a high incidence of SCID (1 in 2, 906 live births) using T-cell receptor excision circle (TREC)-based newborn screening (NBS), while Suliaman et al. reported a high incidence of SCID in the Eastern Province of Saudi Arabia [20,21]. Furthermore, Alnakhli et al. reported an association between PID and secondary malignancies in Saudi patients, highlighting malignancy as an important clinical complication of PID/IEI [22]. Overall, regional studies and newborn screening data support the importance of severely inherited forms in Saudi Arabia. However, these findings provide regional- and cohort-specific insights that cannot be interpreted as national incidence estimates.

Data from regional registries in the Middle East and North Africa have further emphasized the significance of the IEI in populations with high levels of consanguinity. In the Middle East and North Africa (MENA)-IEI registry, 17,120 patients from 22 different countries have been reported, including 502 patients from Saudi Arabia, with parental consanguinity documented in 60.5% of cases. This regional evidence further supports the need for stronger registry-based data and more consistent reporting of PID/IEI across Saudi Arabia and neighboring countries [14]. Overall, the available evidence from Saudi Arabia and the surrounding regions indicates that combined immunodeficiencies, SCID, consanguinity, delays in diagnosis, and severe clinical outcomes are recurring themes in the literature. Nevertheless, the available evidence is limited by the lack of a thorough national registry and the fact that most published data come from specialized tertiary care centers, which may limit the generalizability of the findings, as most published data are derived from referral populations. For contextual purposes, Table 2 summarizes the available Saudi evidence alongside GCC/MENA and international reports; the data are not directly comparable because the underlying studies differ in design, populations, and settings.

## 4. Clinical Presentation, Diagnostic Delay, and Disease Outcomes

### 4.1. Clinical Presentation and Diagnostic Approach

Despite the global variations in the clinical manifestations of PID, recurrent respiratory infections, particularly sinopulmonary and ear infections, are uniformly the most common presentation [32]. Cutaneous and gastrointestinal infections, including diarrhea, abscesses, sepsis, septic arthritis, meningitis, autoimmunity, and malignancies are other well-established clinical manifestations of PID [33]. Therefore, the early diagnosis of PID is critical for reducing infections, complications, morbidity, and hospitalization.

The diagnosis of PIDs is a multistage process comprising history and examination, baseline laboratory tests, baseline immunological tests (e.g., immunoglobulin levels), TREC/KREC newborn screening, and specialized genetic tests, such as high-throughput NGS, whole-exome sequencing (WES), and whole-genome sequencing (WGS) [34]. These advanced testing techniques allow the rapid simultaneous examination of multiple genes, and are preferred for assessing typical and atypical manifestations of heterogeneous PIDs. Although PID manifestations may appear at any age, Al-Saud et al. reported an overall mean age of 17 months for symptom onset and 21.6 months for delay in diagnosis in their Saudi tertiary-care cohort, with earlier symptom onset among patients with combined immunodeficiencies [18]. Globally, a considerable proportion of PID/IEI cases remain unrecognized owing to heterogeneous clinical presentations, limited awareness, and gaps in diagnostic resources, such as limited diagnostic facilities and high costs [16,35]. This highlights the relevance of a delayed diagnosis as an important clinical and healthcare challenge in Saudi Arabia.

### 4.2. Clinical and Healthcare Consequences of Delayed Diagnosis in Saudi Arabia

If PIDs or IEIs are left untreated or unidentified, they may result in recurrent and severe infections, organ damage (to the lungs, liver, or nervous system), autoimmune diseases, and malignancies. Consequently, untreated or undiagnosed PIDs may result in increased mortality, psychological distress, and financial burdens on families. Failure to promptly identify PIDs has serious clinical and public health consequences, including a high risk of recurrent, persistent, and severe infections that lead to irreversible organ damage, chronic lung disease, and long-term morbidity [30]. Many people also develop autoimmunity, inflammatory complications, and malignancies owing to immune dysregulation. Delayed diagnosis increases healthcare utilization through repeated hospitalizations and overuse of antibiotics, while missing early interventions such as immunoglobulin replacement or hematopoietic stem cell transplantation (HSCT) [1]. These results highlight the importance of the prompt identification and precise diagnosis of PIDs. Asediq et al. conducted a cross-sectional study of 51 children with IEI at King Abdullah Specialized Children Hospital, Riyadh, and reported low quality of life and emotional functioning [36]. Similarly, Al-Saud et al. reported that Saudi pediatric patients with PID experienced improved quality of life (QoL) scores after switching from hospital-based intravenous immunoglobulin (IVIG) to home-based 20% subcutaneous immunoglobulin. This finding suggests that treatment delivery models can affect patient burden and daily functioning [37].

In addition to a reduced quality of life, delayed or complicated diagnosis of PID/IEI can lead to severe clinical outcomes, including mortality. In the KFSHRC registry, Al-Saud et al. found that 52 of 502 patients died, yielding an overall mortality rate of 10.3%. Most deaths occurred during childhood, with SCID being the most common diagnosis in deceased patients [18]. Moreover, recent regional data from Saudi Arabia has indicated that pulmonary complications are significant contributors to the clinical impacts of PID/IEI. A retrospective study involving 56 pediatric patients with IEI from southwestern SA found that complicated pneumonia was the most prevalent complication, affecting 85.7% of patients. This study also reported diagnostic delays and the presence of bronchiectasis in some patients, and identified pulmonary complications as the primary cause of mortality. These findings emphasize the importance of early recognition, timely referral, genetic testing, and newborn screening to reduce severe lung-related morbidity and mortality [38].

In Saudi Arabia, several factors may complicate the early diagnosis of PIDs, including clinical variability, genetic heterogeneity, high consanguinity, and limited access to specialized diagnostic testing. A national survey protocol was developed to assess awareness of PID diagnosis among physicians working in primary healthcare centers (PHCCs) [17,33]. Educational sessions or seminars for PHCC physicians and the general population, along with the increased availability of screening and specialized tests (e.g., genetic testing with NGS, WES, and WGS), may facilitate the early detection and management of PIDs, thereby reducing the risk of recurrent infections, hospitalizations, morbidities, and healthcare costs. Despite these recognized challenges, data on the broader healthcare burden of PID/IEI in Saudi Arabia remain limited. Few studies have measured hospitalization rates, healthcare utilization, treatment costs, long-term morbidity, and family financial burdens. Current evidence indicates that delayed diagnosis may lead to recurrent infections, complications, hospital visits, morbidity, and higher healthcare expenses. Robust multicenter and registry-based studies are needed to assess the national epidemiology and clinical impact of PID/IEI accurately [18,30].

## 5. Treatment and Management

Management of PIDs requires multidisciplinary care involving immunologists, hematologists, infectious disease specialists, clinical geneticists, and other relevant specialists. Treatment for PIDs may include lifelong immunoglobulin replacement therapy to reduce the frequency of recurrent infections and improve patient QoL, antibiotic prophylaxis and infection management, antifungal or antiviral therapy to prevent or treat opportunistic infections, cytokine therapy, targeted therapies for complications, and nutritional and supportive care [39]. For selected PID/IEI disorders, definitive or disease-modifying treatments include HSCT, gene therapy, and enzyme replacement therapy (ERT) [27]. Overall, IEI/PID management is prolonged and expensive.

Immunoglobulin replacement therapy is the standard when clinically indicated, particularly for antibody deficiencies. Al-Saud et al. conducted an open-label, single-center study of 24 patients with PIDs from the Immunodeficiency Clinic or Immunology Post-HSCT Clinic at KFSHRC between July 2018 and August 2021, comparing the efficacy of IVIG with that of 20% subcutaneous immunoglobulin (SCIG). They reported an improvement in QoL among children with PID after switching from hospital-based IVIG to home-based 20% (SCIG) [37]. The Saudi healthcare system is currently equipped to provide therapeutic management for PIDs, primarily through HSCT. There are four leading centers in Saudi Arabia specializing in HSCT, of which the KFSHRC in Riyadh, established in 1984, was the first HSCT unit in Saudi Arabia [40]. The other three HSCT units were the Prince Sultan Military Medical City in Riyadh (established 1996), KFSHRC in Jeddah (established 2001), and King Fahad Specialist Hospital in Dammam (established 2010). In partnership with germ-free laboratories, KFSHRC has advanced gene therapy initiatives in Saudi Arabia [41]. However, advanced therapies for certain PID/IEI conditions, such as gene therapy and enzyme replacement therapy (ERT), are not widely available in routine clinical practice. There is a scarcity of published data on the use, accessibility, outcomes, and long-term implementation of these therapies in Saudi Arabia. Furthermore, the evidence regarding the application of ERT in PID/IEI treatment in Saudi Arabia remains limited. Most Saudi studies continue to emphasize the importance of immunoglobulin replacement therapy, HSCT, antimicrobial prophylaxis, and supportive care [19]. Published evidence in Saudi Arabia on the routine availability, use, and outcomes of gene therapy and ERT remains limited. Further research on the use and outcomes of ERT for selected PID/IEI disorders is therefore required.

## 6. Healthcare System Challenges

In Saudi Arabia, PID/IEI care is affected by several system-level challenges, including delayed recognition, restricted access to specialized diagnostics, referral inefficiencies, incomplete registry coverage, and gaps in long-term care coordination. Although the published Saudi literature provides important clinical insights, there is a lack of comprehensive system-level evidence. Overall, these challenges should be viewed as emerging themes identified in current studies rather than fully quantified national issues [14,18,28].

### 6.1. Physician Awareness

Limited awareness among non-specialist healthcare providers may delay the recognition of PID/IEI, particularly in patients who experience recurrent infections, failure to thrive, poor response to antibiotics, or a positive family history. Formal nationwide assessments of primary care physicians’ awareness in Saudi Arabia are limited. Current evidence indicates knowledge gaps among medical trainees and in primary care, highlighting the need for targeted education, practical referral criteria, and early-recognition tools [28,42].

A major barrier to the early detection of PID/IEI in Saudi Arabia is the limited knowledge of these conditions among some healthcare worker groups, particularly PHCC physicians. Although this can delay diagnosis, no studies have yet formally assessed the current knowledge of PHCC physicians. The literature reports that even in the USA, only 32% of physicians regularly diagnose, treat, report, or refer patients with PIDs [17]. In this context, a national survey protocol (KFSHRC/MOH) was established to systematically assess PIDs awareness among kingdom-wide PHCC physicians [33]. Studies in the literature have reported low knowledge of PIDs among medical students in Saudi Arabia [42]. Alghamdi et al. conducted a cross-sectional study involving 387 medical students in the 3rd, 4th, 5th, and 6th years medical students at four universities in Riyadh, Saudi Arabia [42], reporting that 83% of medical students possessed below-average PID knowledge.

Public awareness has also been studied in settings where medical professionals have limited knowledge of PIDs and the relevant diagnostic protocols, although the available evidence remains limited. In this context, Esmaeel et al. conducted a cross-sectional study of 528 participants aged 20–30 years in Arar, Saudi Arabia, to assess the public awareness of PIDs in children [43]. They reported that 9.1% of the participants knew about children with PID. The participants were also aware of the symptoms of PIDs in children. Among the participants, the most common conditions were delayed growth (47%), chronic diarrhea (34.1%), sinusitis (33.3%), and otitis media (25.8%). Moreover, 66% of the participants knew that blood tests were essential for diagnosing PIDs, while 50% recognized the need for genetic testing for this purpose [43]. Interestingly, this study reported high public awareness of PIDs, which may be attributed to the participants’ education levels, with 88% having higher education. Therefore, to address misdiagnosis and delayed recognition, there is a need for curriculum development and training initiatives focused on PID/IEI awareness, screening, diagnostic evaluation, and treatment pathways. These initiatives should specifically target PHCC physicians, pediatricians, medical trainees, and other frontline healthcare providers who are likely to encounter patients before their referral to immunology services. Implementing structured educational activities for these groups may enhance the early suspicion of PID/IEI and decrease delays in referral to specialized immunology services. Table 3 summarizes the diagnostic and referral approaches for suspected PID/IEI based on the available Saudi registry, newborn screening, awareness, and current IEI diagnostic recommendations [8,18,20,33,38,44].

### 6.2. Referral Systems

Early referral is critical for patients with suspected PID/IEI, as timely access to clinical immunology, genetic testing, infection management, and definitive therapies can reduce complications and improve patient outcomes [38]. However, in Saudi Arabia, specialized services are predominantly located in large tertiary centers, potentially limiting access to patients residing far from these major tertiary-care centers. Although direct national data on PID/IEI referral pathways are limited, the concentration of published studies in major centers indicates that referral patterns and regional access may affect diagnosis and reporting. Establishing clear referral criteria, developing regional care networks, and enhancing coordination among primary care, pediatrics, hematology, infectious diseases, genetics, and immunology services may facilitate earlier detection and improve the continuity of care.

### 6.3. Cost, Family Burden, and Patient Education

PID/IEI commonly result in significant long-term clinical, psychological, and financial burdens. Many patients require repeated investigations, hospital admissions, antimicrobial therapy, immunoglobulin replacement, genetic testing, and, in severe cases, HSCT [18,39]. Despite these challenges, there is a lack of Saudi-specific data regarding treatment costs, healthcare utilization, and insurance-related issues. Future studies should therefore aim to assess healthcare utilization and the economic burden to support effective resource planning and ensure equitable access to specialized care.

Patient and family education both play a critical role in enhancing treatment adherence, preventing infections, facilitating early recognition of warning signs, and supporting long-term follow-up. This is especially important in Saudi Arabia, as inherited forms of PID/IEI can affect several family members and may require non-directive genetic counseling, family screening, and information about clinically available options [18,45]. Patient support programs, structured counseling, and accessible educational materials may help reduce psychological distress and promote care engagement. Notably, Saudi studies have reported reduced QoL among children with IEI and demonstrated improved QoL scores following home-based SCIG therapy [36,37].

### 6.4. Public Health and Policy Implications

Key public health priorities include earlier diagnosis, enhanced registry coverage, expanded newborn screening for severe forms of SCID, and better access to specialized diagnostic and treatment services. Saudi studies have further demonstrated the potential benefits of newborn screening for SCID, with international evidence indicating that early SCID detection leads to better outcomes [20,46,47]. Additionally, Saudi Arabia has established national newborn screening programs for various inherited and metabolic disorders, which could serve as a foundation for expanding future screening priorities [48].

## 7. Research Gaps and Future Directions

Despite the increasing recognition of PID/IEI and the Health Sector Transformation Program under Saudi Vision 2030, which aims to provide efficient, sustainable, and competitive healthcare facilities in Saudi Arabia, several evidence gaps remain. First, most published data were derived from single-center or tertiary-care studies, which limits our understanding of the national prevalence and regional variation [18,23,38]. Second, data on diagnostic delays, healthcare utilization, treatment costs, quality of life, and long-term outcomes were limited [18,36,45]. Third, there is a need for broader genetic and genotype–phenotype studies, particularly given the high rate of consanguinity and the likelihood of autosomal recessive forms [18,23,38].

Overall, future research should prioritize multicenter and registry-based studies that monitor clinical phenotypes, genetic diagnoses, treatment pathways, complications, survival, quality of life, and healthcare utilization. These data would facilitate a more precise estimation of the national epidemiology and clinical impact and help guide newborn screening policies, referral pathways, specialized service planning, family counseling, and equitable access to advanced diagnostics and therapies.

## 8. Conclusions

The PID/IEI represents an important, but incompletely defined, healthcare impact in Saudi Arabia. Existing studies indicate that consanguinity, severe inherited forms such as SCID, and diagnostic delays contribute to the clinical impact of the condition, while reliance on tertiary care data limits national interpretation. The national epidemiology and clinical impact remain incompletely characterized owing to the limited registry coverage, regional data gaps, insufficient evidence on hospitalizations, financial burden on the healthcare system, long-term outcomes, and quality of life.

Improving PID/IEI care in Saudi Arabia will require an integrated approach that includes national registry development, expanded newborn screening for disorders such as SCID, improved primary care physician awareness, clearer referral pathways, broader access to immunological and genetic testing, family counseling, and stronger multicenter research. These priorities align with the broader objectives of the Healthcare Sector Transformation Program, particularly its emphasis on improving prevention, healthcare quality, access, and system efficiency.

## Figures and Tables

**Table 1:** Contextual Overview of Major IEI Categories According to IUIS.

Major IEI Category	Brief Clinical Relevance	Examples
Immunodeficiencies affecting cellular/humoral immunity	Early-onset, often severe and relevant to SCID	SCID CID
Combined immunodeficiencies with syndromic features	Immunodeficiency with developmental anomalies	DiGeorge syndrome Wiskott–Aldrich syndrome
Predominant antibody deficiencies	Often later diagnosis and recurrent sinopulmonary infections	XLA CVID Selective IgA deficiency
Immune dysregulation	Autoimmunity, lymphoproliferation, inflammation	ALPS IPEX CTLA4 deficiency
Phagocytic defects	Recurrent bacterial/fungal infections, abscessus	CGD Leukocyte adhesion deficiency
Defects in intrinsic and innate immunity	Susceptibility to specific infection	TLR3 pathway defects
Autoinflammatory disorders	Recurrent systemic inflammation and fever syndromes	FMF CAPS
Complement deficiencies	Recurrent infections and autoimmunity	C3 deficiency C2 deficiency C1q deficiency
Bone marrow failure	Cytopenia and immune dysfunction	Dyskeratosis congenita GATA2 deficiency
Phenocopies of IEI	Acquired or somatic conditions resembling IEI	Somatic ALPS Autoantibody-related syndrome

Abbreviations: ALPS: Autoimmune lymphoproliferative syndrome; CAPS: Cryopyrin-associated periodic syndrome; C1q: Complement component 1q; C2: Complement component 2; C3: Complement component 3; CGD: Chronic granulomatous disease; CID: Combined immunodeficiency; CTLA4: Cytotoxic T-lymphocyte-associated protein 4; CVID: Common variable immunodeficiency; FMF: Familial Mediterranean fever; GATA2: GATA-binding protein 2; IEI: Inborn errors of immunity; IgA: Immunoglobulin A; IPEX: Immune dysregulation, polyendocrinopathy, enteropathy, X-linked; IUIS: International Union of Immunological Societies; SCID: Severe combined immunodeficiency; TLR3: Toll-like receptor 3; XLA: X-linked agammaglobulinemia.

**Table 2:** Selected Reported Findings on PID/IEI in Saudi Arabia with Contextual GCC/MENA and International Data.

Indicator	Saudi Arabia	GCC/MENA	Global	Interpretation and Key Limitations
Reported Prevalence	7.2 per 10^5^ children [18]	7–11.9 per 10^5^ [23]	2.5 per 10^5^ persons [24]	Saudi prevalence estimates are based mainly on tertiary-care cohorts; population-level prevalence remains uncertain.
Mean Age at Diagnosis	3.25 years [23]	3.5–3.8 years [23]	Variable. 3.3–4.8 years [25]	Saudi data consistently indicate diagnostic delay, although evidence is derived mainly from referral centers.
Most Common PID/IEI type	Combined Immunodeficiencies	No combined GCC study on PIDs. Cellular and Humoral Immunodeficiencies in Kuwait [26] Phagocyte Disorders in Oman [27] Predominantly antibody deficiency in Qatar [28] Combined immunodeficiencies with associated or syndromic features in the UAE [29]	Antibody deficiencies or Humoral IEIs [16]	Combined immunodeficiencies/SCID in Saudi cohorts but may reflect consanguinity, referral bias, and pediatric case mix
Diagnostic Delay	1.8 years [19]	1.5–2.3 years [23]	1.0–3.7 years [30]	Diagnostic delay is consistently reported, although estimates vary across study populations and healthcare settings
SCID incidence	1 in 2906 live births [20]; 19 per 10^5^ live births in Eastern Province [21]	4.5 per 100,000 Omani live births [31]	Variable depending on the screening program	Regional newborn-screening studies provide valuable data, but national incidence remains uncertain.
Mortality and severe outcomes	10.3% overall mortality in the KFSHRC PID registry [18], 11/18 deaths in Eastern Province SCID cohort [21]	69.4% mortality in Oman SCID cohort [31]	Variable	Available mortality data are limited to selected cohorts and should not be interpreted as national estimates.
Registry coverage	Hospital-based registry data are available; a population-based national registry is limited.	Limited (hospital-based)	Established in high-income countries	Hospital-based registries provide important data but remain insufficient for comprehensive national surveillance.
Newborn Screening	Pilot programs in hospitals	Rare	Implemented in high-income countries	Broader implementation of SCID newborn screening is a key policy priority. Pilot programmes demonstrate feasibility, but nationwide implementation remains incomplete.
Genetic Testing	Genetic testing is available in specialist centers	Limited	Available in high-income settings	Access to genetic testing has expanded, although availability and published national data remain limited.

Abbreviations: GCC: Gulf Cooperation Council; IEI: Inborn errors of immunity; KFSHRC: King Faisal Specialist Hospital & Research Centre; MENA: Middle East and North Africa; PID: Primary immunodeficiency; SCID: Severe combined immunodeficiency; UAE: United Arab Emirates.

**Table 3:** Proposed Diagnostic and Referral Approach for Suspected PID/IEI in Saudi Arabia.

Step	Clinical Trigger or Action	Responsible Team	Saudi Relevance/Practical Gap
1	Clinical suspicion: Recurrent respiratory, ear, sinus, skin, or gastrointestinal infections; failure to thrive; poor response to antibiotics; sepsis; unusual infections; autoimmunity; malignancy; positive family history	PHCC physician Pediatrician	Early clinical suspicion is crucial in children born to consanguineous parents or with a family history of PID/IEI because these factors increase the likelihood of an inherited immune disorder.
2	Initial evaluation: Clinical history, family history, physical examination, CBC with differential, lymphocyte count, and immunoglobulin levels	PHCC physician Pediatrician	Basic, widely available tests can facilitate early recognition prior to specialist referral, even in healthcare centers lacking immunology services.
3	Screening for severe early-onset disease: TREC/KREC newborn screening where available; prompt immunological evaluation when SCID is clinically suspected	Neonatologist pediatrician Laboratory team	Early detection of SCID is supported; however, TREC/KREC newborn screening nationally, and nationwide coverage has not been yet documented, representing an opportunity for further national evaluation
4	Referral to specialized care when PID/IEI is suspected	Pediatrician Clinical immunologist	Standardized, clearly defined referral criteria may help reduce diagnostic delays and subsequent complications.
5	Specialized diagnosis: Flow cytometry, lymphocyte subsets, vaccine antibody responses, functional tests, NGS/WES/WGS	Clinical immunologist Geneticist Laboratory team	Advanced diagnostic testing is primarily available in tertiary care centers, and turnaround times may delay critical, time-sensitive decisions such as HSCT timing.
6	Management: Antimicrobial prophylaxis, IVIG/SCIG, infection control, HSCT for selected severe cases, supportive care	Clinical immunologist Infectious disease Transplant team	Effective management requires ongoing multidisciplinary coordination and access to specialized services; Related-donor selection in consanguineous families requires careful clinical and genetic evaluation.
7	Family care: Genetic counseling, family screening, targeted newborn screening for affected families	Genetic counsellor Clinical immunologist Pediatrician	Particularly important in Saudi Arabia, given high rates of consanguinity and familial recurrence, underscoring the need for cascade screening among relatives.
8	Long-term follow-up and registry reporting	Multidisciplinary team	Essential for tracking outcomes and complications over time; the absence of a comprehensive national registry remains a major evidence gap that limits accurate estimation of disease impact at the population level.

Abbreviations: CBC: Complete blood count; HSCT: Hematopoietic stem cell transplantation; IEI: Inborn errors of immunity; IVIG: Intravenous immunoglobulin; KREC: Kappa-deleting recombination excision circle; NGS: Next-generation sequencing; PHCC: Primary Health Care Center; PID: Primary immunodeficiency disorder; SCID: Severe combined immunodeficiency; SCIG: Subcutaneous immunoglobulin; TREC: T-cell receptor excision circle; WES: Whole-exome sequencing; WGS: Whole-genome sequencing.

## Data Availability

No new data were created or analyzed in this study. Data sharing is not applicable to this article.
